# Nurses' Perspectives and Understanding of Sarcopenia in a Tertiary Care Hospital

**DOI:** 10.1155/2024/9106500

**Published:** 2024-09-12

**Authors:** Kanjana Khuankaew, Panita Limpawattana, Manchumad Manjavong, Nutwara Saengwijit, Khanyanut Ojongpien, Prapassawan Tanlawan

**Affiliations:** ^1^ Srinagarind Hospital Faculty of Medicine Khon Kaen University, Khon Kaen 40002, Thailand; ^2^ Internal Medicine Department Faculty of Medicine Khon Kaen University, Khon Kaen 40002, Thailand

## Abstract

**Background:**

Sarcopenia is prevalent in older adults. It is essential for nurses to sustain updated information regarding the knowledge of sarcopenia, particularly in relation to approaches to diagnosing and addressing sarcopenia. However, there are limited studies examining the attitudes and awareness of nurses in relation to this condition.

**Objective:**

To assess the attitude and knowledge of nurses regarding sarcopenia and correlation between positive attitude and scores on knowledge.

**Materials and Methods:**

An electronic survey was carried out among the nurses of the Faculty of Medicine, Khon Kaen University, Thailand, during November 2022 and January 2024. This survey encompassed questionnaires aimed at evaluating the nurses' perspectives and understanding of sarcopenia. The participants were invited to complete the survey, and they were returned to the researchers for analysis.

**Results:**

A total of 231 nurses were recruited (response rate 46.2%). Only 58.4% of them were familiar with “sarcopenia,” while only 16% had confidence in diagnosing it. For general knowledge, they achieved a total score of 19/30 (63.3%). They demonstrated proficiency in “etiology” (75%), while their performance was fair in “management and prevention” (62.5%) and low in “terminology & importance” (50%) and “diagnosis” (50%). The correlation between positive attitude and knowledge on sarcopenia was 0.22 (*p* < 0.05).

**Conclusion:**

Nurses exhibited a reduced awareness regarding “sarcopenia.” Their comprehension about terminology, diagnosis, prevention, and management of this condition was limited. There was a weak correlation between positive attitude and knowledge. The findings emphasize the essentiality of augmenting the educational programs to enhance the recognition of sarcopenia among nurses.

## 1. Introduction

Sarcopenia, a phenomenon characterized by the decline in muscle mass and function that occurs with advancing age, has been found to have associations with various comorbidities including chronic inflammatory disease, malnutrition, and immobility [1–3]. Sarcopenia exhibits a robust correlation with various detrimental health consequences such as physical disability, diminished quality of life, and escalated mortality. It is also linked to heightened advancement of cardiovascular diseases, prolonged hospitalization, falling, occurring of fractures, and perioperative complications [3–5]. Age, gender, and the level of physical activity are significant risk factors of sarcopenia. The prevalence of sarcopenia varied based on diagnostic criteria and population being studied. Normally, it was observed that approximately 10–16% of older patients experienced sarcopenia [6]. According to a systematic analysis, it was found that 25% of individuals of African descent were affected, with 55.7% of them being women [7]. Moreover, the prevalence of sarcopenia was notably higher among older patients who were admitted to the hospital, particularly in the department of neurological rehabilitation where it reached approximately 48% [8]. Additionally, among older patients above the age of 75 who suffered from hip fractures, the prevalence of sarcopenia was found to be 53% [9]. The diagnosis of sarcopenia primarily focuses on the assessment of muscle strength, muscle mass, and physical performance. The treatment approach for sarcopenia, in addition to addressing the underlying contributing factors, encompasses exercise therapy, specifically resistance training, and nutritional therapy. Currently, there is no specific approved medication for sarcopenia [1–3, 6, 10]. In 2016, an individual International Statistical Classification of Diseases and Related Health Problems code (M62.84) was assigned to this condition to enhance awareness and understanding among healthcare professionals [1]. Numerous studies and working groups are currently being conducted worldwide to promote research and therapeutic trials for sarcopenia. These include the Asian Working Group on Sarcopenia [1], the Korean Working Group on Sarcopenia (KWGS) [11], the European Working Group on Sarcopenia for Older People (EWGSOP) [12], and the Australian and New Zealand Society for Sarcopenia and Frailty Research (ANZSSFR) [13].

It is crucial for nurses to remain well informed about the understanding of sarcopenia, especially concerning current methods of diagnosis and intervention for treating sarcopenia. This knowledge is necessary in order to adopt a proactive approach to screening and to establish a paradigm of tertiary prevention. Moreover, an interprofessional team is required to aid sarcopenic patients or those at risk in recovering, rebuilding, and rehabilitating their muscle mass and strength [14, 15]. Despite the growing body of research dedicated to sarcopenia and the publication of multiple guidelines for its management, the present literature on nurses' attitudes and knowledge regarding sarcopenia remains scarce [16–19]. The existing research conducted in Australia, New Zealand, Netherlands, Brazil, and China has demonstrated that there is a limited understanding of sarcopenia among nurses [16–19]. However, these nurses exhibited a positive attitude towards sarcopenia [16]. Furthermore, it has been observed that the retention of knowledge about sarcopenia following a professional development event for a period of more than 6 months was also limited [19]. These findings highlight a significant gap in practice when it comes to providing appropriate management for sarcopenia in older adults. Additionally, there is a lack of research on the attitude and knowledge regarding sarcopenia among nurses in the Southeast Asia. In order to develop an effective approach and management in this area, it is essential to understand the current perspective and knowledge among nurses. Hence, the aim of this study was to determine the attitude and level of knowledge about sarcopenia and explore the correlation between positive attitude and knowledge among nurses who provided care for older patients in a tertiary care hospital.

## 2. Methods

### 2.1. Populations and Study Design

This survey-based analysis included nurses who cared for older patients at Srinagarind University Hospital, Thailand, locating in the northeast Thailand during November 2022 and January 2024. The participants who did not return the electronic questionnaires were excluded.

### 2.2. Instrument Development

The electronic questionnaires were designed to assess the attitudes and knowledge about sarcopenia among nurses, in accordance with the Asian Working Group for Sarcopenia's 2019 consensus update on sarcopenia diagnosis and treatment (AWGS 2019) [1].

An expert committee from Khon Kaen University, comprising a geriatrician, gerontologic nurse, and a physician nutrition specialist, conducted a thorough review on sarcopenia, focusing on its definition, significance, etiology, diagnostic approaches, treatment, and preventive measures. These questionnaires consisted of two parts: the first part collected demographic information and explored prior experience in the fields of geriatric medicine, nutrition, or sarcopenia training, while the second part assessed attitudes towards sarcopenia (which included 9 items) and general knowledge about sarcopenia (which encompassed 30 items, further categorized into 14 items related to terminology and importance, 4 items pertaining to etiology, 8 items focusing on diagnosis, and 8 items addressing management and prevention). A five-point Likert scale was employed to evaluate the attitudes regarding sarcopenia, while binary responses of “yes” or “no” were necessitated for the knowledge assessment questionnaires associated with sarcopenia.

### 2.3. Process

The electronic questionnaires were made accessible to the eligible participants for the purpose of data collection. In the beginning, the details of the questionnaires clarified the purpose of the investigation, the expected advantages, and the intended recipients of the statements. The nurses were instructed to conscientiously complete the questionnaire according to their own judgment. Measures were taken to guarantee the privacy of the respondents, and no compensation was offered. Following that, the completed surveys were handed back to the researchers.

### 2.4. Ethical Aspects

The study, under reference number HE 651502, was granted approval by the Institutional Review Board of Khon Kaen University. The project, which encompassed survey procedures, was found to be exempt from review by the Ethics Committee. Consequently, the necessity for informed consent was waived.

### 2.5. Statistical Analysis

The content validity of the questionnaires was assessed by another geriatrician and two physician nutrition specialists using the scale-content validity index (S-CVI) [20]. Face validity was also assessed among the targeted participants, comprising 10 nursing staff working at the faculty of Medicine, Khon Kaen University, Thailand, to assess the comprehensibility of the questionnaires. Assessment of the questionnaire's internal consistency was conducted by calculating Cronbach's alpha. The value extends from zero to one with higher values indicating that the items are measuring a similar dimension. The acceptable reliability of each scale is 0.7 or above [21]. Moreover, correlation coefficients were computed for the item-item in order to ascertain the internal correlation strength of each item. It provides an evaluation of item redundancy. It is recommended that the average of interitem correlation falls between 0.15 and 0.50 for a group of items, indicating a suitable level of homogeneity among the items while still possessing distinct variability to prevent them from being identical to one another [22]. Demographic data were analyzed using descriptive statistics and presented as percentages, means, and standard deviations. In instances where the data did not follow a normal distribution, medians and interquartile ranges were used instead. Since the distribution of the data was not normal, the Spearman rank correlation was employed to ascertain the correlation between positive attitude and total scores on sarcopenia knowledge. The determination of statistically significant differences was based on a *p* value of less than 0.05. In order to consolidate responses expressing positive attitude with the statements, the individual responded a favorable disposition regarding sarcopenia, as evidenced by her/his strong agreement or agreement with statements that reflect a positive attitude on sarcopenia such as “I have heard about sarcopenia,” “I know about how to diagnose sarcopenia,” and “every hospitalized patient who is expected to stay over a week should have physical therapy consultation.” This individual would score one if she/he responded others, she/he would score zero. In contrast, if the individual expressed strong agreement or agreement with negative attitude regarding sarcopenia such as “In clinical practice, prevention of common medical diseases with severe complication such as diabetes mellitus, hypertension is more important than prevention of sarcopenia,” “Sarcopenia lies beyond the realm of my expertise, as it lacks any association with my patients,” “The patient without muscle atrophy is likely to have normal muscle mass,” she/he would receive a score of zero, while other responses would yield a score of one. The imputation method was used to deal with missing data by calculating the median of the existing observations and replacing the missing values with these figures [23]. The data analysis was performed using STATA version 10.0 (StataCorp, College Station, TX, USA).

## 3. Results

A total of 231 electronic questionnaires were successfully obtained from a sample of 500, resulting in a response rate of 46.2%, and the characteristics of studied populations are demonstrated in Table 1. The median age and years of practice was 37 and 14 years, respectively, with female predominate. Nurses from noninternal medicine and orthopedic and rehabilitation ward were most of the participants. About 42% of them received training in geriatric medicine.

### 3.1. Attitude regarding Sarcopenia among Nursing Staff

Attitude on selected issue on sarcopenia among nursing staff is shown in Table 2. Nearly 60% of the participants demonstrated familiarity with the term “sarcopenia,” while a mere 16% possessed the confidence to diagnose it. Approximately 60% of the participants directed their attention towards the prevention of metabolic disorders, such as diabetes, rather than the prevention of sarcopenia. However, the majority of the participants expressed disagreement towards the notion that sarcopenia was beyond their level of expertise, whereas the minority of the participants agreed that muscle atrophy and normal muscle power shared similarities with sarcopenia. Roughly 75% of the participants opined that any patient admitted to the hospital with an expected duration of stay exceeding one week should receive consultation for physical therapy. Furthermore, 41.5% of the participants held the belief that the treatment of sarcopenia required the expertise of a healthcare professional specializing in that particular area.

### 3.2. Validity and Reliability of the Instrument to Determine the Knowledge of Sarcopenia among Nursing Staff

The scale-content validity index (S-CVI) of this instrument was 0.94. For the face validity, most of the respondents demonstrated a clear understanding of the questions, although some were deemed overly challenging. Drawbacks of the existing questionnaires were recorded. The internal consistency for each domain analysis is demonstrated in Table 3. There were 0.80 for terminology and importance (10 items), 0.83 for etiology (4 items), 0.87 for diagnosis (8 items), and 0.83 for management (8 items) which indicated a good level of internal consistency. The average of the internal correlations among the items for terminology and importance was 0.33, for etiology was 0.48, diagnosis was 0.44, and for management was 0.38.

### 3.3. General Knowledge and Factors Associated with the Numbers of Corrected Answers on Sarcopenia among Nursing Staff

The participants successfully attained a cumulative score of 19 out of 30, equivalent to 63.3%. They showcased their expertise in the field of “etiology” by achieving a score of 75%. However, their performance in the domains of “management and prevention” was considered satisfactory, with a score of 62.5%. Conversely, their proficiency in the areas of “terminology & importance” and “diagnosis” was found to be suboptimal, with scores of 50% in both categories (Table 4).

### 3.4. Correlation of Positive Attitude and Scores on Sarcopenia Knowledge

**Figure 1:**
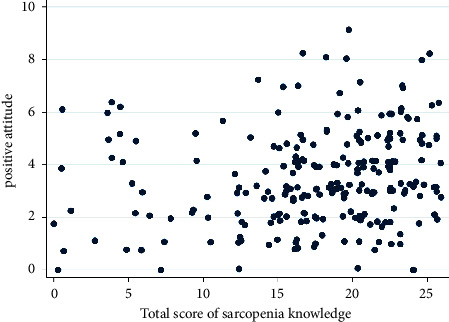
Scatter plot of the correlation between positive attitude and scores on sarcopenia knowledge.

There was a weak correlation between positive attitude and scores on sarcopenia knowledge with a Spearman rank correlation coefficient of 0.22 (95% confidence interval 0.09–0.35, *p* < 0.05).  Figure 1 shows a scatter plot of this correlation.

## 4. Discussion

This was the first study that assessed the attitude and knowledge about sarcopenia among nursing staff who provided care for geriatric patients in the Southeast Asian region. The findings from our survey revealed that although around 60% of the nursing staff had familiar with the term “sarcopenia,” they possessed limitation in comprehending its concept, diagnosis, management, and prevention. This finding was not unexpected, given that earlier research yielded similar results [16–18]. For instance, a qualitative study conducted among primary care nurses in Brazil reported that their familiarity with sarcopenia screening in older adults was rudimentary and fragmented [17]. Another survey study carried out among nursing staff from secondary and tertiary care hospitals in China indicated that they possessed minimal knowledge about sarcopenia, although they exhibited a positive outlook on this matter [16]. In line with a scoping review that explored awareness of sarcopenia among healthcare professionals, which included studies conducted in Australia, Netherlands, and Brazil but none from Asia, and the results indicated that existing reports provided limited awareness and understanding of sarcopenia [18]. The potential explanations can be elucidated by the fact that the term “sarcopenia” was relatively novel compared to other geriatric syndromes, such as delirium, resulting in a limited awareness of sarcopenia. However, there has been a recent surge in research in this field, and specific guidelines have been developed in various regions, including Asia [1, 11–13]. Despite this, the practical implementation of these guidelines in clinical settings remains restricted. For example, the hand dynamometer for sarcopenia screening and the bioelectrical impedance analysis (BIA) for measurement of muscle mass are not available widely in healthcare settings of Thailand even in tertiary care hospitals. In addition, time constraints of diagnostic measures were limited due to the high volume of the patients. Furthermore, the awareness and knowledge about sarcopenia among other healthcare professionals, particularly physicians who often collaborated with nursing staff, are also limited. Consequently, a lack of motivation in this domain has been identified as the primary barrier to sarcopenia diagnosis and management [18]. Secondly, there existed a lack of focus on emphasizing nursing curricula and continuous educational programs to enhance the knowledge of nursing staff as demonstrated from our finding. Only a small portion of participants received training specifically focused on sarcopenia though around 40% have participated in training in the field of geriatric medicine. Although the nursing staff exhibited a lesser degree of awareness and a restricted scope of knowledge regarding sarcopenia in our findings, we observed a positive correlation between a positive attitude and knowledge pertaining to sarcopenia such as approximately 75% of them believed that early rehabilitation for hospitalized patients at risk of sarcopenia was of utmost importance. This finding aligned with previous studies [16] highlights the inclination to actively seek further knowledge on sarcopenia and prioritize its impact in professional pursuits.

There were certain limitations associated with this study. Firstly, the sample size employed in the study was relatively small due to the low response rate for the questionnaires. This could be attributed to the utilization of an online self-report questionnaire, which might have elicited socially desirable responses. Consequently, it could be inferred that the participants exhibited limited awareness and insufficient knowledge regarding sarcopenia. Secondly, the generalizability of the findings might be restricted, as the study only included nursing staff from a single tertiary care hospital. Further research involving multilevel healthcare facilities would be beneficial in gaining a deeper insight into the attitudes and level of knowledge regarding sarcopenia among nursing staff. Moreover, conducting an additional analysis concerning the attitudes and knowledge of sarcopenia among nurses who acquired their licensure prior to and after the inclusion of sarcopenia in the ICD-10 classification system would be beneficial. Thirdly, the utilization of this quantitative methodology might constrain the results, as it might not fully capture the attitudes of the participants towards sarcopenia. To gain a more comprehensive understanding of the barriers hindering the development of a positive attitude and knowledge towards sarcopenia, further research employing a larger sample size and a mixed-method design inclusive of qualitative data collection is recommended.

The findings of this study imply that specialized training on sarcopenia, which could be initiated during undergraduate education and extended into postgraduate and continuing medical education, can be implemented through different methods, such as online learning and information platforms, interactive interventions, or other innovative approaches in conjunction with traditional didactic teaching methods, with the aim of enhancing nurses' awareness and knowledge of sarcopenia. In addition, the establishment of national policies and specific clinical practice guidelines for screening, diagnosing, and managing sarcopenia by using available assessment instruments and practical management processes would serve to support nursing staff in increasing the awareness, education, and expertise of all healthcare professionals regarding sarcopenia [16, 19, 24].

## 5. Conclusion

The term “sarcopenia” was familiar to approximately 60% of the nursing staff; however, their awareness and understanding of the concept, diagnosis, management, and prevention of sarcopenia were limited. A positive correlation was observed between a positive attitude and knowledge regarding sarcopenia. These findings highlight the necessity for policymakers and healthcare professionals to collaborate to develop clinical practice guidelines that incorporate an available assessment tool and enhance medical education programs, starting from undergraduate studies and continuing through postgraduate and continuing education. This collaborative effort is essential for increasing awareness and knowledge regarding sarcopenia among nursing staff.

## Figures and Tables

**Table 1:** Characteristics of the studied population.

Variables	*n* = 231	
Age (years); med (IQR 1, 3)	37	(30, 48)
Female; *n* (%)	209	(90.5)
Duration of practice (years); med (IQR 1, 3)	14	(7, 24)
Working place; *n*(%)		
Outpatient clinic	14	(6.1)
Internal medicine ward	19	(8.2)
Orthopedic and rehabilitation ward	27	(11.7)
Noninternal medicine and orthopedic and rehabilitation ward	53	(22.9)
Academic unit	6	(2.6)
Special ward	26	(11.3)
Critical care ward	47	(20.3)
Emergency	17	(7.4)
Operation unit	15	(6.5)
Others	7	(3.0)
Clinical experience of training; *n* (%)		
Geriatric medicine	98	(42.4)
Nutrition	33	(14.3)
Sarcopenia	9	(3.9)

*Note. n*: numbers of participants, med: median, IQR: interquartile.

**Table 2:** Attitude on the selected issue on sarcopenia among nursing staff.

Statement	Level of agreement (*N* = 211)				
	Strongly agree	Agree	Neutral	Disagree	Strongly disagree
I have heard about “sarcopenia” before	60 (25.9%)	75 (32.5%)	65 (28.1%)	20 (8.7%)	11 (4.8%)
I know how to diagnose sarcopenia	6 (2.6%)	31 (13.4%)	100 (43.3%)	51 (22.1%)	43 (18.6%)
In clinical practice, prevention of common medical diseases with severe complication such as diabetes mellitus, hypertension is more important than prevention of sarcopenia	55 (23.8%)	60 (35.9%)	64 (27.7%)	32 (13.9%)	20 (8.7%)
Sarcopenia lies beyond the realm of my expertise, as it lacks any association with my patients	9 (3.9%)	16 (6.9%)	55 (23.8%)	71 (30.8%)	80 (34.6%)
Sarcopenia is like cachexia or starvation	18 (7.8%)	38 (16.5%)	83 (35.9%)	64 (27.7%)	28 (12.1%)
The patient without muscle atrophy is likely to have normal muscle mass	13 (5.6%)	48 (20.8%)	98 (42.4%)	44 (19.1%)	28 (12.1%)
The patient with normal muscle power is likely to have normal muscle mass	27 (11.7%)	81 (35.0%)	79 (34.2%)	32 (13.9%)	12 (5.2%)
Every hospitalized patient who is expected to stay over a week should have physical therapy consultation	85 (36.8%)	88 (38.1%)	38 (16.5%)	16 (6.9%)	4 (1.7%)
The treatment of sarcopenia necessitates a complex approach, requiring the expertise and involvement of specialized healthcare professionals	22 (9.5%)	74 (32.0%)	84 (36.4%)	33 (14.3%)	18 (7.8%)

**Table 3:** Knowledge domain analysis on sarcopenia.

Domain	Items	Statement	Crobach
(A) Terminology and importance	1	Sarcopenia has been classified as a “medical condition” in accordance to the International Classification of diseases, Tenth Revision (ICD-10)	0.80
	2	Individuals with obesity are at risk of developing sarcopenia	
	3	Sarcopenia is a natural process of aging	
	4	Sarcopenia tends to be more prevalent in men than in women	
	5	The normal range of muscle mass differs between the two sexes	
	6	The normal range of handgrip strength differs between the two sexes	
	7	Individuals with sarcopenia face an increased susceptibility to falls	
	8	Individuals with sarcopenia are at a heightened risk of cardiovascular disease	
	9	Individuals with sarcopenia face an decrease in quality of life	
	10	Individuals with sarcopenia are at an increased risk of hospital-acquired infection	

(B) Etiology	11	Typically, sarcopenia is commonly associated with increasing age	0.83
	12.	Sarcopenia is prevalent in patients with chronic diseases such as diabetes mellitus, chronic obstructive pulmonary disease, chronic heart failure, chronic liver disease, and chronic kidney disease	
	13	Malnutrition is a significant factor in the development of sarcopenia	
	14.	The classification of sarcopenia can be delineated into acute sarcopenia (<6 months) and chronic sarcopenia (persisting for 6 months or longer)	

(C) Diagnosis	15	The recommendation of the Asian working group for sarcopenia in 2019 proposes the use of appendicular skeletal mass as an indicator of muscle mass in the diagnostic evaluation of sarcopenia	0.87
	16	Muscle strength and physical performance serve as indicators for evaluating the severity of sarcopenia	
	17	Calf circumference can be used to screen sarcopenia	
	18	The SARC-F and SARC-calf questionnaires have been recommended by the Asian working group for sarcopenia in 2019 for screening sarcopenia	
	19	Gait speed serves as indicators for evaluating the extent of sarcopenia	
	20	Handgrip strength serves as indicators for evaluating the extent of sarcopenia	
	21	Assessment of muscle mass can be performed using dual-energy X-ray absorptiometry (DXA)	
	22	Assessment of muscle mass can be performed using bioelectrical impedance analysis (BIA)	

(D) Management and prevention	23	Treatment of choice for sarcopenia is pharmacological intervention	0.83
	24	Anabolic agents, like testosterone, represent the primary pharmacotherapy for this condition	
	25	Aerobic exercise is an effective way to increase muscle mass	
	26	Adequate energy and high-quality protein can effectively enhance muscle mass	
	27	The combination of exercise and nutritional intervention enhances muscle mass and strength more effectively than either approach alone	
	28	Avoiding alcohol consumption helps in delaying the decline in muscle mass	
	29	Avoiding smoking helps in delaying the decline in muscle mass	
	30	Sarcopenia is a preventable condition	

**Table 4:** Total score on sarcopenia knowledge.

Topics	No. of correct answers; median (IQR 1, 3)	No. of items	(%); median (IQR 1, 3)
(A) Terminology and importance	5 (5, 8)	10	50 (50, 80)
(B) Etiology	3 (3, 4)	4	75 (74, 100)
(C) Diagnosis	4 (1, 6)	8	50 (12.5, 75)
(D) Management and prevention	5 (5, 6)	8	62.5 (62.5, 75.0)
Total score	19 (15, 22)	30	63.3 (50, 73.3)

## Data Availability

The data that support the findings of this study are available from the corresponding author, [PL], upon reasonable request.
